# Supplementary material for: Session Rating of Perceived Exertion Is a Valid Method to Monitor Intensity of Exercise in Adults with Acute Burn Injuries
Source: Eur Burn J. 2025 Jan 30;6(1):4. doi: 10.3390/ebj6010004 (PMC11843880; doi:10.3390/ebj6010004)
Supplement: Supplementary file 1 [file ebj-06-00004-s001.zip › ebj-3342782-supplementary.pdf]

Supplementary Table S1: The univariate associations of predictor variables with session RPE

Predictor variable	*β*	$SE_β$	R^2	*p value*
**HR average for session**	0.013	0.013	0.019	0.343
**Peak HR**	0.015	0.011	0.035	0.195
**Peak HR as a percentage of age predicted HR max**	-0.017	0.009	0.068	0.071
**Average RPE for session**	1.146	0.107	0.709	**<0.001**
**BLa Change (mmol/L)**	0.101	0.075	0.037	0.186
**Post exercise BLa (mmol/L)**	0.149	0.074	0.079	0.051
**BLa percentage change from baseline**	0.002	0.001	0.031	0.230

Abbreviations: HR = heart rate, RPE = ratings of perceived exertion, BLa = blood lactate.
B = unstandardized regression coefficient: SE_B = standard error of the coefficient: R^2 = coefficient of determination.

Supplementary Table S2: The univariate associations of predictor variables on training load (session RPE × session duration).

Predictor variable	*β*	$SE_β$	R^2	*p value*
**HR average for session**	0.693	0.577	0.030	0.236
**Peak HR**	1.076	0.479	0.097	**0.029**
**Peak HR as a percentage of age predicted HR max**	-0.866	0.385	0.097	**0.029**
**Average RPE for session**	40.184	6.362	0.459	**<0.001**
**BLa change (mmol/L)**	5.858	3.218	0.066	0.075
**Post exercise BLa (mmol/L)**	7.071	3.193	0.094	**0.032**
**BLa percentage change from baseline**	0.092	0.054	0.057	0.097

Abbreviations: HR = heart rate, RPE = ratings of perceived exertion, BLa = blood lactate.
B = unstandardized regression coefficient: SE_B = standard error of the coefficient: R^2 = coefficient of determination.
